# Multi-omics revealed the mechanism of feed efficiency in sheep by the combined action of the host and rumen microbiota

**DOI:** 10.1016/j.aninu.2024.04.009

**Published:** 2024-04-27

**Authors:** Guangchen Zhou, Junda Li, Xuhui Liang, Bohua Yang, Ximeng He, Hongyu Tang, Hongran Guo, Gongwei Liu, Wenyuan Cui, Yulin Chen, Yuxin Yang

**Affiliations:** Key Laboratory of Animal Genetics, Breeding and Reproduction of Shaanxi Province, College of Animal Science and Technology, Northwest A&F University, Yangling 712100, China

**Keywords:** Residual feed intake, Sheep, Microbial composition, Rumen proteome, Liver transcriptome, Metabolite

## Abstract

This study was conducted to investigate potential regulatory mechanisms of feed efficiency (FE) in sheep by linking rumen microbiota with its host by the multi-omics analysis. One hundred and ninety-eight hybrid female sheep (initial body weight = 30.88 ± 4.57 kg; 4-month-old) were selected as candidate sheep. Each test sheep was fed in an individual pen for 60 days, and the residual feed intake (RFI) was calculated. The ten candidate sheep with the highest RFI were divided into the Low-FE group, and the ten with the lowest RFI were divided into the High-FE group, all selected for sample collection. The RFI, average daily gain and average daily feed intake were highly significantly different between the two experimental groups (*P* < 0.05). Compared with Low-FE group, the insulin-like growth factor-1 and very low-density lipoprotein in serum and the propionate in rumen significantly increased in High-FE group (*P* < 0.01), but the acetate:propionate ratio in rumen significantly decreased in High-FE group (*P* = 0.034). Metagenomics revealed *Selenomonas ruminantium, Selenomonas* sp. and *Faecalibacterium prausnitzii* were key bacteria, and increased abundance of the genes encoding the enzymes for cellulose degradation and production of propionate in High-FE group. The results of proteomics and section showed the rumen papilla length (*P* < 0.001) and expression of carbonic anhydrase and Na^+^/K^+^-ATPase were significantly higher in High-FE group (*P* < 0.05). On the other hand, the acetyl-CoA content significantly increased in the liver of High-FE group (*P* = 0.002). The relative expression levels of insulin-like growth factor-1 and apolipoprotein A4 genes were significantly up-regulated in the liver of High-FE group (*P* < 0.01), but relative expression level of monoacylglycerol O-acyltransferase 3 gene was significantly down-regulated (*P* = 0.037). These findings provide the mechanism by which the collaborative interaction between rumen microbiota fermentation and host uptake and metabolism of fermentation products impacts feed efficiency traits in sheep.

## 1 Introduction

Livestock products are important sources of high-quality protein for the human diet. Growing food insecurity in conjunction with an increasing human population challenges the supply of feeding materials and increases feeding costs. High feed cost continues to fuel interest in alternative approaches to improve livestock production efficiency (Na and Guan, 2022). Improving the feed efficiency (FE) of livestock is not only an important method for profitable animal husbandry but also the core goal of animal husbandry science. The mode of intensification production has been widely used in the sheep industry. Feeding in the same environment under the same management practices improves the productivity of feeding sheep. However, some individuals show higher FE under this feeding mode, which can provide a potential method for the selection of sheep and the development of feed additives.

The rumen serves as a bioreactor that can decompose natural fibre by rumen microbiota (Xue et al., 2022a); fibre decomposition by microbial enzymes produces volatile fatty acids (VFA) for utilization by herbivorous animals. The short chain volatile fatty acids (SCFA) produced in the rumen can meet 70% of the total energy requirements of ruminants (Bergman, 1990). Thus, previous studies have proposed that the community structures of the rumen microbiota could be one of the main influencing factors for ruminant FE (Li et al., 2019a; Xue et al., 2022b). However, it is difficult to achieve long-term and stable improvement of production performance only by transplanting microbiota (Cox et al., 2021; Zhou et al., 2018). Additionally, it has been found that the host may play a regulatory role in the rumen microbiota (Li et al., 2019b). However, there is still a lack of a systematic summary of the impact mechanisms of hosts and microbiota on FE. Importantly, in most studies, dairy cows or beef cows are selected as experimental objects. Studies on the FE of sheep are underestimated, and most studies reporting the rumen microbiota relative to the FE of sheep are at the genus level (McLoughlin et al., 2020; Zhang et al., 2021). This limitation hinders the development and application of microbial preparations for sheep. In addition, causal studies on the interaction between the rumen microbiota and host and their impact on FE in sheep remain limited (Zhang et al., 2019, Zhang et al., 2021). This falls short of meeting the requirements for livestock production. Therefore, systematically exploring the causal relationships between microbiota and host factors and their impact on the phenotype of feed utilization efficiency in sheep, as well as identifying key microbial species and predictive markers, is crucial for the development of the sheep industry.

We hypothesize that high FE sheep may possess regulatory mechanisms, enhancing FE through interactions between specific rumen microbial species and host metabolic processes, especially crude fibre utilization. In this study, we performed metagenomics, transcriptomics, proteomics, and analyses of metabolites in serum and rumen samples from sheep with significantly different residual feed intakes (RFI) to explore the influencing mechanism of FE and further determine specific rumen microbial and metabolite features, which can be used as predictive markers in selecting high FE in sheep. Furthermore, the current study will be able to support breeding selection of sheep and the development of bacterial preparations to improve productivity of the feeding industry.

## 2 Material and methods

### 2.1 Animal ethics statement

The use of animals and all experimental protocols (protocol number: 100403) were authorized by the Institutional Animal Care and Use Committee of Northwest A&F University (Yangling, Shaanxi, China).

### 2.2 Experimental animals and design

**Fig. 1:**
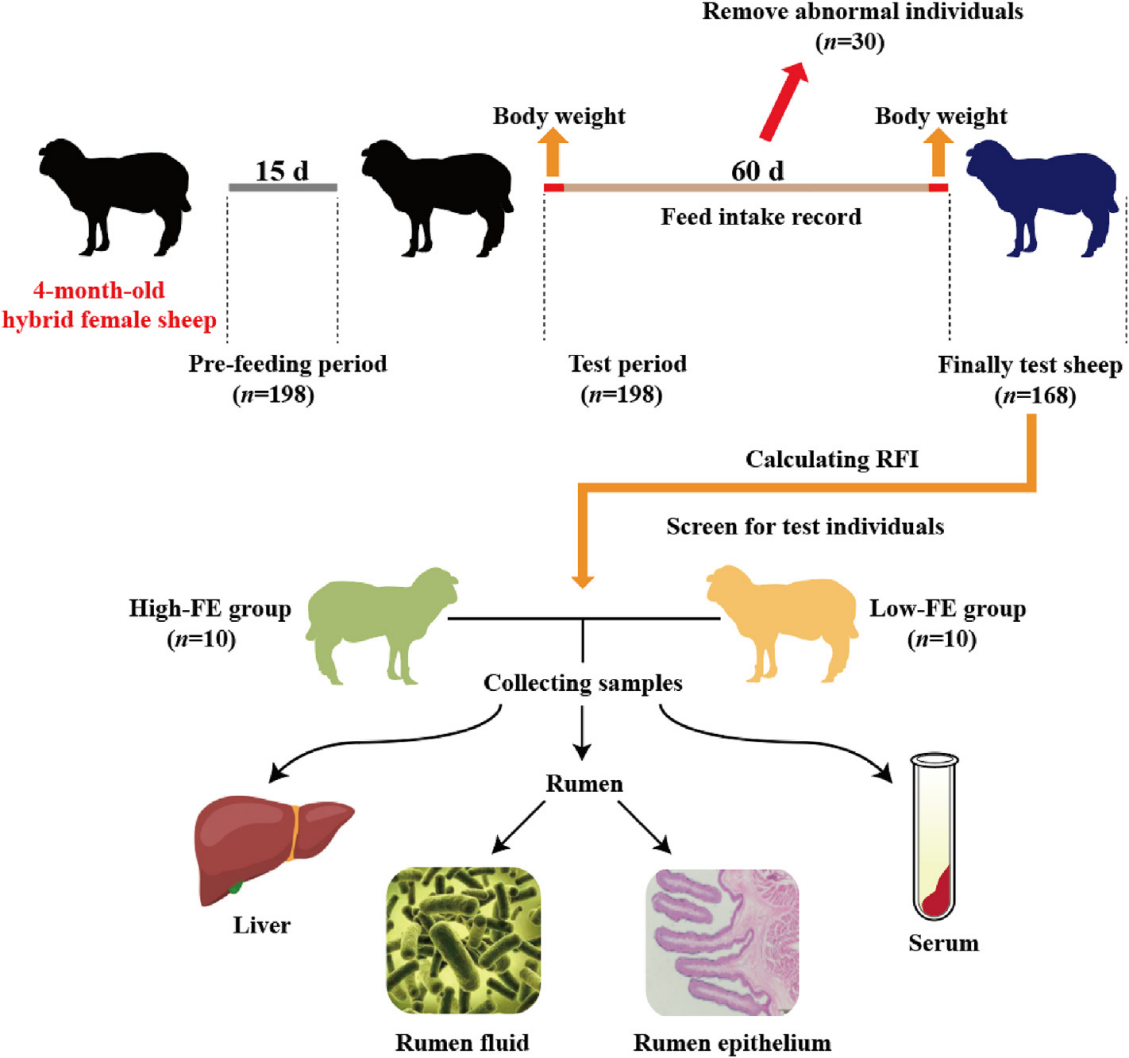
The experimental design depicting collection of samples and data analysis. RFI = residual feed intake; Low-FE = extreme individual sheep with the highest RFI; High-FE = extreme individual sheep with the lowest RFI.

This study was conducted at a sheep breeding farm in Huan County, Qingyang city, Gansu Province, China (36.39′ N, 107.46′ E). One hundred and ninety-eight hybrid female sheep (Suffolk × Hu sheep, initial body weight = 30.88 ± 4.57 kg) were selected as candidate sheep. Each test sheep was fed in an individual pen (6 m × 4 m) with automatic water supply equipment. All test sheep were fed the same total mixed ration twice a day at 06:00 and 18:00, ensuring more than 20% remaining feed in the trough every day. Composition and nutrient levels of the experimental diet are shown in Table 1. Before feeding the test sheep, the feed for each sheep was accurately weighed, and the remaining feed of each sheep was collected and weighed at 05:00 and 17:00 every day before feeding. The feed intake was calculated before the morning feeding. Faeces were removed daily during the study to minimize the release of harmful gases and the accumulation of pathogenic microorganisms. The experiment was divided into two phases: the pre-feeding period (15 d) and the test phase (60 d). During the experiment, we observed the health condition of all test sheep every day and eliminated the test sheep with poor physical condition over time (*n* = 30). The design of the study is presented in Fig. 1.

**Table 1:** Composition and nutrient levels of the experimental diet (DM basis, %).

Ingredients	Content	Nutrient levels^2^	Content
Oat grass	12.43	ME^3^, MJ/kg	13.75
Corn silage	29.00	CP	16.40
Corn	19.34	EE	3.00
Wheat	20.72	Ash	9.58
Soybean meal	5.68	ADF	16.80
Cottonseed meal	4.00	NDF	27.80
Corn gluten meal	7.00	Lignin	4.30
Limestone	0.83	Ca	0.88
Premix^1^	1.00	P	0.48
Total	100.00		

ME = metabolic energy; CP = crude protein; EE = ether extract; NDF = neutral detergent fibre; ADF = acid detergent fibre.

1 One kilogram premix contained the following: vitamin A 600,000 IU, vitamin D 200,000 IU, vitamin E 2000 IU, Fe 5.5 g, Zn 5 g, Cu 1 g, Mn 3 g.

2 All data were measured except for ME.

3 Metabolic energy was calculated as follows (Menke and Steingass, 1988): ME (MJ/kg DM) = 13.97 – 0.0127 × ADF (g/kg DM) + 0.0165 × EE (g/kg DM) – 0.0057 × Ash (g/kg DM).

### 2.3 Sample collection

Analyses performed on feed samples were as follows: the crude protein content of the diet was tested using the Kjeldahl method (AOAC, 2000; method 976.05); the ether extract content of the diet was tested using the Soxhlet extractor method (AOAC, 2005; method 920.39); the Van Soest detergent fire analysis method was used for the determination of neutral detergent fibre (NDF), acid detergent fibre (ADF) and lignin contents (Van Soest et al., 1991); the calcium content was tested using potassium permanganate titration (AOAC, 1990; method 985.35); the phosphorus content was determined using colorimetry with molybdenum yellow (AOAC, 1990; method 986.24). The production performance of the test sheep is shown in Table 2.

**Table 2:** The production performance of test sheep.

Item	Initial body weight,	Final body weight,	ADG,	ADFI,	RFI
	kg	kg	kg/d	kg/d	
All test sheep (*n* = 168)	30.94 ± 4.756	39.25 ± 4.931	0.14 ± 0.036	2.41 ± 0.317	0.00 ± 0.298
Other test sheep (*n* = 148)	30.90 ± 4.871	39.17 ± 4.997	0.14 ± 0.035	2.43 ± 0.289	0.02 ± 0.208

ADG = average daily gain; ADFI = average daily feed intake; RFI = residual feed intake.

Daily feed intake was recorded for every individual sheep (*n* = 168) throughout the experimental period to measure average daily feed intake (ADFI). The body weight of each test sheep was recorded before feeding on the morning of days 0 and 60 of the normal feeding phase, and the average daily gain (ADG) of each test sheep was calculated. The ADFI, weight and ADG data of the test sheep were used to calculate the RFI by the following formula:

*Y*_*i*_ = *β*_0_ + *β*_1_ (ADG_*i*_) + *β*_2_ (MBW^0.75^_*i*_) + *e*_*i*_.

Where *Y*_i_ is the expected ADFI (kg/d) in the *i*th animal; *β*_0_ represents the regression intercept; ADG_i_ is the average daily gain; *β*_1_ represents the extent to which ADG affects feed intake; *β*_2_ represents the extent to which average interim metabolic weight affects feed intake; and *e*_i_ represents RFI. The MBW is given by the formula: MBW = [1/2 × (FBW_i_ + IBW_i_)], where FBW is the final BW, and IBW is the initial BW. The ten candidate sheep with the highest RFI were divided into the Low-FE group, and the ten with the lowest RFI were divided into the High-FE group, all selected for sample collection. Blood (20 mL) was collected by jugular venipuncture and transferred into a serum separator tube. Blood samples in the serum separator tube were centrifuged at 3000 × *g* for 20 min. The isolated serum was stored at −20 °C for analysis of biochemical indicators. All selected extreme RFI value individual sheep were euthanized by a qualified operator after prohibiting feeding and drinking. The enterocoel was then opened, and the rumen, reticulum, omasum, abomasum, and duodenum were separated with a suture line to avoid reflux of digesta among adjacent regions. Afterwards, relevant digesta samples were collected and homogenized separately. In addition, an extra 20 mL of rumen fluid was strained through a four-layer sterile gauze. Ruminal pH was measured immediately after sampling using a pH meter (Mettler Toledo Instruments Co. Ltd., Shanghai, China). All samples were placed in a cryotube and placed in liquid nitrogen for storage. The Van Soest detergent fire analysis method was used for the determination of NDF contents of digesta in the rumen and reticulum. The liver and rumen wall of the ventral sac were removed and rinsed with phosphate buffered saline (PBS) to remove impurities. The tissue was flash frozen in liquid nitrogen approximately 10 min after stunning and euthanizing. In addition, an extra 2 cm × 2 cm of rumen tissue was fixed in 4% paraformaldehyde, embedded in paraffin, sectioned and stained with haematoxylin and eosin (HE) for examination. The slices were observed under an optical microscope at a magnification of 40 × (Olympus BX-51; Olympus Corporation, Tokyo, Japan), and the length and width of the rumen papillae were measured using the Image-Pro Express image analysis system (Image-Pro Plus 6.0, Media Cybernetics, Silver Spring, MD, USA).

### 2.4 Measurements of biochemical parameters

One gram of liver was ground and dissolved in 10 mL of exclusive extract solution. The supernatant was used for biochemical parameters after centrifugation. The biochemical parameters in serum and liver, including insulin-like growth factor-1 (IGF-1), acetyl-CoA, β-hydroxybutyrate, insulin, very low-density lipoprotein (VLDL), triglycerides and glucose (GLU) were measured in accordance with specific reagent kit protocols (Beijing Sino-UK Institute of Biological Technology, Beijing, China). Serum IGF-1 and insulin contents were determined using a microplate reader (DR-200BS, Wuxi Hiwell-Diatek Instruments Co., Ltd., Wuxi, China). Acetyl-CoA, β-hydroxybutyrate, VLDL, triglycerides, and GLU contents were determined using an automated biochemical analyser (Mindray BS-420 Chemical Analyser, Shenzhen Mindray Biomedical Electronics Co., Ltd., Shenzhen, China).

### 2.5 VFA and NH_3_–N concentration measurement

The concentrations of VFA were determined using gas chromatography (Agilent 7820A, Santa Clara, CA, USA) with a capillary column (AE-FFAP, 30 m × 0.25 mm × 0.33 μm; ATECH Technology Co., Ltd., Lanzhou, China). The procedure was as follows: rumen fluid samples were centrifuged at 16,000 × *g* for 10 min at 4 °C. Two millilitres of the supernatant were mixed with 25% metaphosphoric acid (400 μL). After standing at 4 °C for 4 h, the mixture was centrifuged at 16,000 × *g* for 10 min at 4 °C. Two hundred microlitres of the supernatant was taken as an aliquot and mixed with 200 μL of crotonic acid (10 g/L), followed by filtration through a 0.45 μm filter. The injector and detector temperatures were set at 200 and 250 °C, respectively. The column temperature was increased from 45 to 150 °C at a rate of 20 °C/min and maintained for 5 min. The NH_3_–N concentrations were determined using methods described by Broderick and Kang (1980).

### 2.6 Metagenomics analysis in rumen fluid samples and data analysis

#### 2.6.1 DNA extraction and library construction

Total genomic DNA was extracted from rumen fluid samples using the E.Z.N.A. Soil DNA Kit (Omega Bio-Tek, Norcross, GA, USA) according to the manufacturer's instructions. The concentration and purity of extracted DNA were determined by TBS-380 and NanoDrop 2000, respectively. A paired-end library was constructed using NEXTflexTM Rapid DNA-Seq (Bioo Scientific, Austin, TX, USA). Paired-end sequencing was performed on an Illumina NovaSeq 6000 (Illumina Inc., San Diego, CA, USA) at Majorbio Bio-Pharm Technology Co., Ltd. (Shanghai, China) using NovaSeq Reagent Kits.

#### 2.6.2 Bioinformatics analysis

The sequences obtained by sequencing were subjected to quality filtering and modification. The clean reads were mapped to the *Ovis aries* reference genome using BWA (version 0.7.9a) to identify and remove the sheep host-originated reads (Li and Durbin, 2009). These high-quality reads were then assembled into contigs using MEGAHIT (version 1.1.2) (Li et al., 2015). Contigs with lengths over 300 bp were selected to predict open reading frames (ORF) using MetaGene (Noguchi et al., 2006). A nonredundant gene catalogue was constructed using CD-HIT (Fu et al., 2012). After quality control, reads were mapped to the nonredundant gene catalogue with 95% identity using SOAPaligner (version 2.21) (Li et al., 2008). Representative sequences of the nonredundant gene catalogue were annotated based on the NCBI NR database (access date: 20200604) using Diamond (version 0.8.35) for taxonomic annotations (Buchfink et al., 2015). Kyoto Encyclopedia of Genes and Genomes (KEGG) annotation was conducted against the KEGG database (version 9.4.2). Carbohydrate-active enzyme annotation was conducted using hmmscan against the CAZy database.

### 2.7 Transcriptomics analysis of liver samples and data analysis

#### 2.7.1 RNA isolation and purification

Total RNA was extracted from the tissue using TRIzol Reagent according to the manufacturer's instructions (Invitrogen, Carlsbad, CA, USA), and genomic DNA was removed using DNase I (Takara Bio Inc., Shiga, Japan). Then, RNA quality was determined by a 2100 bioanalyzer (Agilent Technologies, Santa Clara, CA, USA) and quantified using an ND-2000 (NanoDrop Technologies, Wilmington, DE, USA).

#### 2.7.2 cDNA library construction and bioinformatics analysis

The RNA-Seq transcriptome library was prepared following the TruSeq RNA Sample Preparation Kit from Illumina (Illumina Inc., San Diego, CA, USA) using 1 μg of total RNA. After quantification by TBS380, the paired-end RNA-sequencing library was sequenced with the Illumina NovaSeq 6000 sequencer (2 × 150 bp read length).

The raw paired end reads were trimmed and quality controlled by SeqPrep and Sickle with default parameters. Then clean reads were separately aligned to reference genome with orientation mode using HISAT2 software (Kim et al., 2015). The mapped reads of each sample were assembled by StringTiein a reference-based approach (Pertea et al., 2015). The expression level of genes was calculated according to the transcripts per million reads (TPM) method (Trapnell et al., 2010). The differentially expressed genes (DEG) were identified by the DESeq2 method (Love et al., 2014). Genes with a fold change (FC) > 2 and *P*-value < 0.05 were selected, and the enrichment of differentially expressed genes was analysed using KEGG pathway analysis.

### 2.8 Total protein extraction, reductive alkylation and digestion

The maximum test sample per time for TMT proteomics was sixteen, so eight rumen epithelial tissues were randomly selected from the High-FE group and Low-FE group as samples to avoid experimental error (Li et al., 2020). The samples in the frozen state were removed and placed on ice. An appropriate amount of protein lysate (8 mol/L urea, 1% sodium dodecyl sulfate) was added. The treated mixture was sonicated for 2 min at a low temperature and allowed to lyse for 30 min. After centrifugation at 12,000 × *g* at 4 °C for 30 min, the concentration of the protein supernatant was determined by the bicinchoninic acid (BCA) method with a BCA Protein Assay Kit (Pierce, Thermo, USA). Protein samples (100 μg) were added to triethylammonium bicarbonate buffer (TEAB). Then, tris (2-carboxyethyl) phosphine (TCEP) was added to a final concentration of 10 mmol/L and reacted for 60 min at 37 °C. Iodoacetamide (IAM) was added to a final concentration of 40 mmol/L and reacted for 40 min at room temperature in the dark. A certain percentage (acetone:sample [vol/vol] = 6:1) of precooled acetone was added to each sample and allowed to settle for 4 h at −20 °C. After centrifugation at 10,000 × *g* for 20 min, the sediment was collected, and 100 μL of 100 mmol/L TEAB solution was added to dissolve the sediment. Finally, the mixture was digested with trypsin overnight at 37 °C and added at a 1:50 trypsin-to-protein mass ratio. The pooled samples were separated by ultra-performance liquid chromatography (UPLC, Acquity, Waters, USA) by an Acquity UPLC BEH C18 column (1.7 μm, 2.1 mm × 150 mm, Waters, USA) to increase the proteomic depth.

The MS/MS search criteria were as follows: methylation of cysteine and the TMT of N-terminus and lysine side chains of peptides as fixed modification, and methionine oxidation as dynamic modifications, respectively. Peptide identification threshold was set at FDR ≤ 0.01, requiring at least one unique peptide for protein identification. Labelled peptides were analysed by online nanoflow liquid chromatography tandem mass spectrometry performed on an Easy-nLC system (Thermo Fisher Scientific, Waltham, MA, USA) connected to a Q_Exactive HF-X instrument (Thermo Fisher Scientific, Waltham, MA, USA) through a nanoelectrospray ionization source. The RAW data files were analysed using Proteome Discoverer (Thermo Scientific, Version 2.4) against the NCBInr database. Proteins with a FC > 1.2 or < 0.83 and a *P*-value < 0.05 were selected.

### 2.9 Statistical analysis

Computational analyses were performed using the following packages available in the R (4.1.3) platform: psych, caret, pROC, and random forest. In the current study, only microbial taxa with a relative abundance higher than 0.01% in at least 70% of individual samples within each extreme individual sheep were reserved for further analysis. The normality of the distribution of data for all data was determined using the Shapiro–Wilk test (*P* > 0.05). Data showing a normal distribution were analysed using a *t*-test, whereas the Wilcoxon test method was used to analyse data that did not show a normal distribution. *P* < 0.05 was considered statistically significant. The random forest package in R was used for the random forest analysis. A 10-fold cross-validation scheme was applied for further evaluation of the model using the caret package in R. The calculation of the area under the curve (AUC) was performed using the pROC package in R. ggplot2, ggtree, pheatmap and ggtreeExtra were used to plot the figures.

## 3 Results

### 3.1 Production performance

The results for the production performance of extreme RFI value individual sheep are shown in Table 3. The initial body weight (IBW) of the two test groups of sheep did not show a significant difference at the start of the test (*P* = 0.913). Among the phenotypes, the ADG, ADFI and RFI were significantly different between the High-FE and Low-FE groups (*P* < 0.05).

**Table 3:** The screening results of the production performance of extreme individual sheep.

Item	Initial body weight,	Final body weight,	ADG,	ADFI,	RFI
	kg	kg	kg/d	kg/d	
Low-FE group	31.15 ± 4.819	37.35 ± 4.714	0.11 ± 0.014	2.73 ± 0.138	0.42 ± 0.081
High-FE group	31.35 ± 2.963	42.30 ± 2.586	0.19 ± 0.020	1.87 ± 0.123	−0.68 ± 0.116
*P-*value	0.913	0.011	<0.001	<0.001	<0.001

ADG = average daily gain; ADFI = average daily feed intake; RFI = residual feed intake; Low-FE = extreme individual sheep with the highest RFI; High-FE = extreme individual sheep with the lowest RFI. *P* < 0.05 was considered statistically significant.

### 3.2 Ruminal fermentation parameters

The concentrations of VFA in the rumen are shown in Table 4. The concentrations of acetate (*P* = 0.040), propionate (*P* = 0.003) and total volatile fatty acids (TVFA) (*P* = 0.034) in the rumen in High-FE group were significantly higher than those in Low-FE group. In addition, we also found that the acetate:propionate ratio (*P* = 0.034) and pH (*P* = 0.047) in the rumen were significantly higher in Low-FE group than in High-FE group.

**Table 4:** Ruminal fermentation parameters between High-FE and Low-FE groups.

Item	Low-FE group (*n* = 10)	High-FE group (*n* = 10)	*P-*value
Acetate, mmol/L	22.07 ± 5.805	26.85 ± 3.312	0.040
Propionate, mmol/L	6.02 ± 1.582	8.42 ± 1.570	0.003
Butyrate, mmol/L	3.05 ± 1.111	3.25 ± 0.700	0.639
Isobutyrate, mmol/L	0.65 ± 0.123	0.54 ± 0.117	0.051
Valerate, mmol/L	0.51 ± 0.144	0.45 ± 0.145	0.385
Isovalerate, mmol/L	0.90 ± 0.171	0.74 ± 0.399	0.256
Total volatile fatty acid, mmol/L	33.14 ± 8.220	40.30 ± 5.215	0.034
Acetate: propionate ratio	3.68 ± 0.466	3.23 ± 0.394	0.034
pH	6.89 ± 0.234	6.64 ± 0.292	0.047
NH_3_–N, mg/dL	8.80 ± 3.423	10.78 ± 4.371	0.275

Low-FE = extreme individual sheep with the highest RFI; High-FE = extreme individual sheep with the lowest RFI; RFI = residual feed intake. *P* < 0.05 was considered statistically significant.

### 3.3 Profiling of the rumen metagenome

**Fig. 2:**
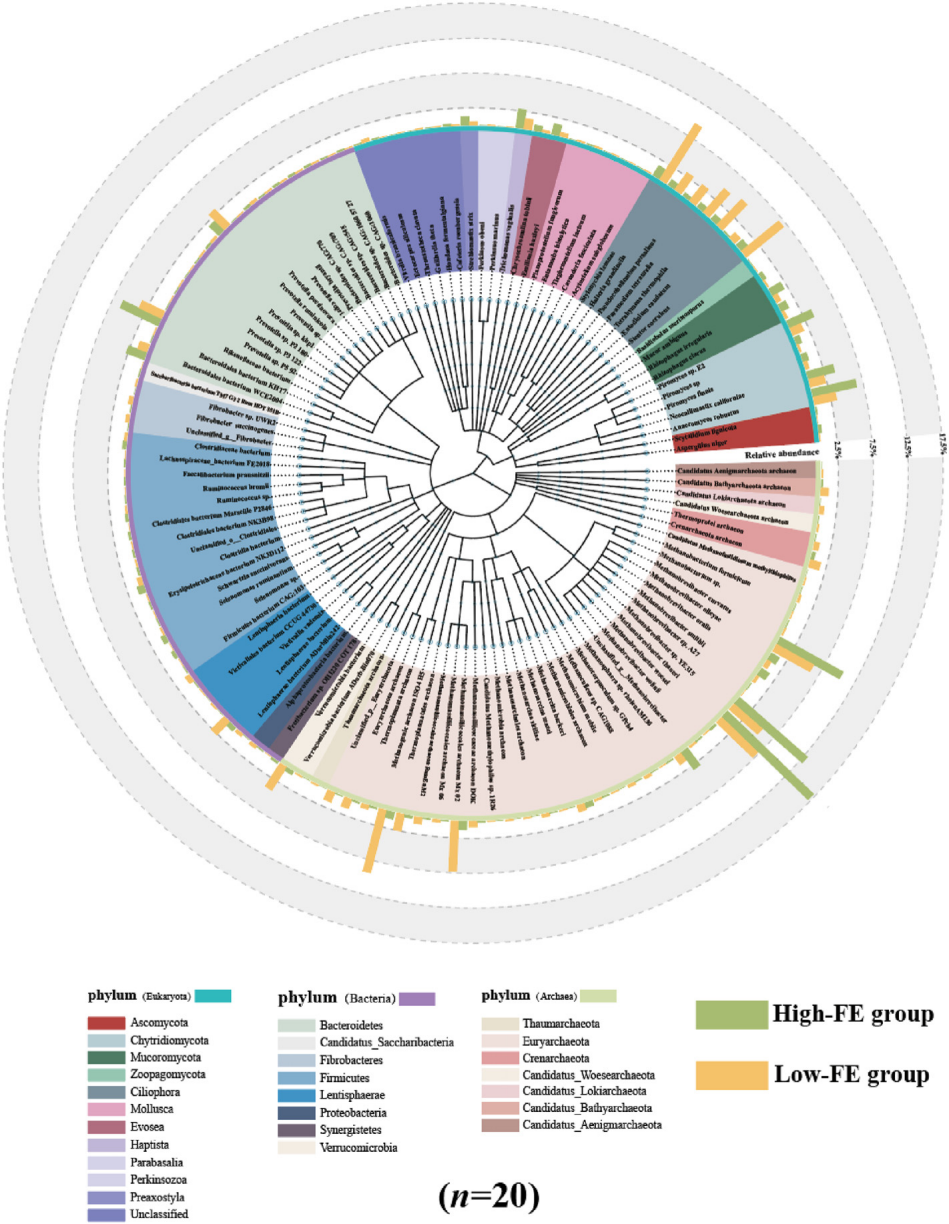
The significantly different microbiota in the rumen between High-FE and Low-FE groups (species level) (*P* < 0.05). Low-FE = extreme individual sheep with the highest RFI; High-FE = extreme individual sheep with the lowest RFI; RFI = residual feed intake. The statistical models were the *t*-test (normally distributed data) and Wilcoxon test (non-normally distributed data).

Metagenome sequencing generated a total of 1,311,015,416 clean reads, and a total of 968,982,500 reads were retained after removing the host genes. After de novo assembly, a total of 15,103,666 contigs were generated (Table S1). The rumen metagenome included 95.79% bacteria (346,286,093 reads), 2.10% eukaryotes (7,606,290 reads), 1.77% archaea (6,413,411 reads) and 0.17% viruses (30,276 reads). Principal coordinate analysis (PCoA) showed separations between the two FE groups based on the microbiota, bacteria, eukaryotes and archaeal species (Fig. S1). The microbiota phyla included Bacteroidetes (46.83%), Firmicutes (37.83%), and Proteobacteria (2.82%) of bacteria, Euryarchaeota (98.11%), Crenarchaeota (0.77%), and Bathyarchaeota (0.31%) of archaea, and Ciliophora (50.74%), Chytridiomycota (5.86%), and Streptophyta (5.85%) of eukaryotes. There were 119 significantly different microbiota species in the rumen of sheep in different groups, including 35 eukaryotic species, 44 bacterial species in 8 bacterial phyla and 40 archaeal species in 7 archaeal phyla (Fig. 2).

### 3.4 Functional profiles of the rumen microbiota

**Fig. 3:**
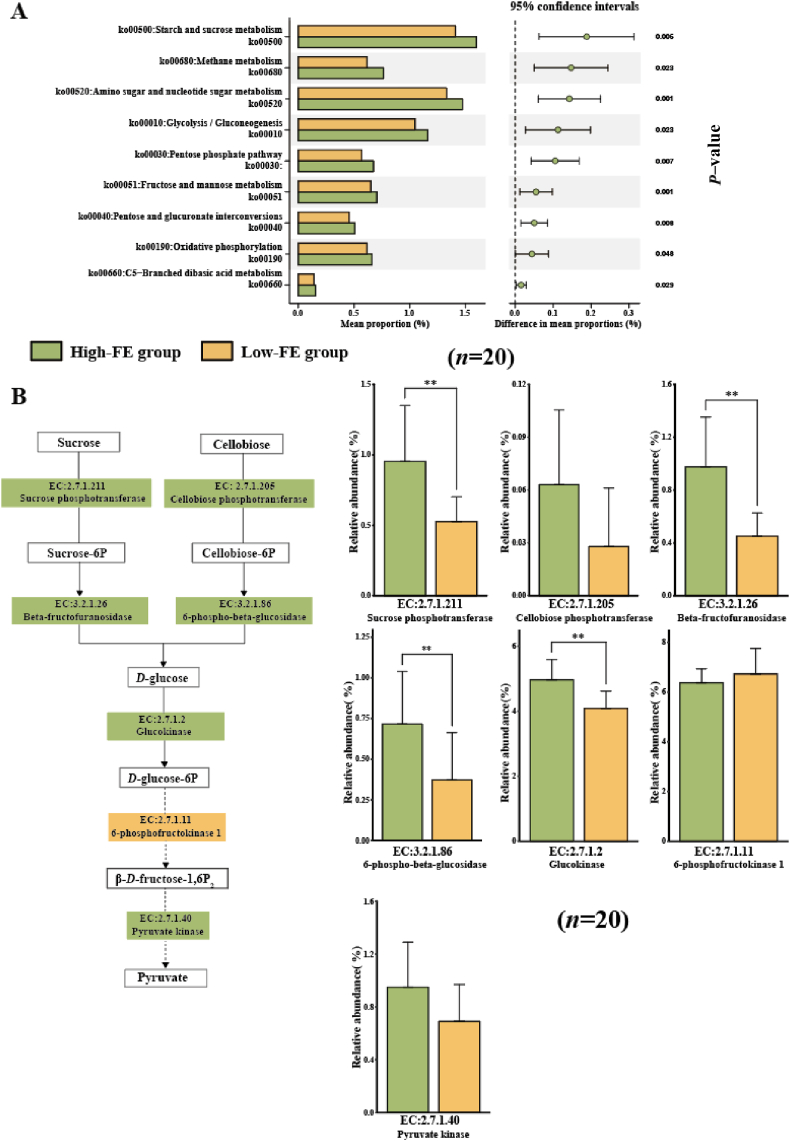
Prediction functions of the rumen microbiota between High-FE and Low-FE groups by KEGG. (A) Significantly different metabolism pathways between High-FE and Low-FE groups. (B) Metabolic pathways involved in cellulose degradation and utilization. Low-FE = extreme individual sheep with the highest RFI; High-FE = extreme individual sheep with the lowest RFI; RFI = residual feed intake. The statistical models were the *t*-test (normally distributed data) and Wilcoxon test (non-normally distributed data). Significant correlations are shown with ∗ (*P* < 0.05) and ∗∗ (*P* < 0.01).

The KEGG database and genes encoding CAZymes were used to further explore the function of the rumen microbiome. The metabolites of the rumen microbiota play an important role in feed digestion in sheep. Thus, the function of the rumen microbiota was focused only on metabolic pathways. We found that nine metabolism pathways were significantly different, including “starch and sucrose metabolism” (ko00500), “methane metabolism” (ko00680), “amino sugar and nucleotide sugar metabolism” (ko00520), “glycolysis/gluconeogenesis” (ko00010), “pentose phosphate pathway” (ko00030), “fructose and mannose metabolism” (ko00051), “pentose and glucuronate interconversions” (ko00040), “oxidative phosphorylation” (ko00190), and “C5-branched dibasic acid metabolism” (ko00660), which were enriched in the rumen of the High-FE group (Fig. 3A).

From the results of the metabolic function of the rumen microbiome, we found that the significantly different pathways were mainly related to carbohydrate metabolism. Crude fibre has been shown to be the main source of carbohydrates for feeding, and we further focused on the genes encoding CAZymes, which are involved in degrading fibre metabolism pathways in the rumen microbiota. The results showed that the relative abundances of genes encoding CAZymes of beta-fructofuranosidase (EC: 3.2.1.26) and 6-phospho-beta-glucosidase (EC: 3.2.1.86) were highly significantly increased in High-FE group (*P* < 0.01). In the “glycolysis/gluconeogenesis” pathway, the gene encoding glucokinase (EC: 2.7.1.2) was significantly higher in the rumen of High-FE group (*P* = 0.002) (Fig. 3B).

For CAZyme profiles, a total of 546 genes encoding CAZymes were identified, including 268 glycoside hydrolases (GH), 18 auxiliary activities (AA), 66 carbohydrate-binding modules (CBM), 16 carbohydrate esterases (CE), 93 glycosyltransferases (GT), and 85 polysaccharide lyases (PL). Among the genes encoding CAZymes of degradation crude fibre, two of GH were enriched in the rumen of High-FE group (GH1 and GH3), while 4 were enriched in the rumen of Low-FE group (GH5, GH10, GH74, and GH141). Xylanase and β-glucosidase were enriched in the rumen of High-FE group, and exoglucanase and endoglucanase were enriched in the rumen of Low-FE group (Table S2).

### 3.5 Associations between microbial species and microbial functions

**Fig. 4:**
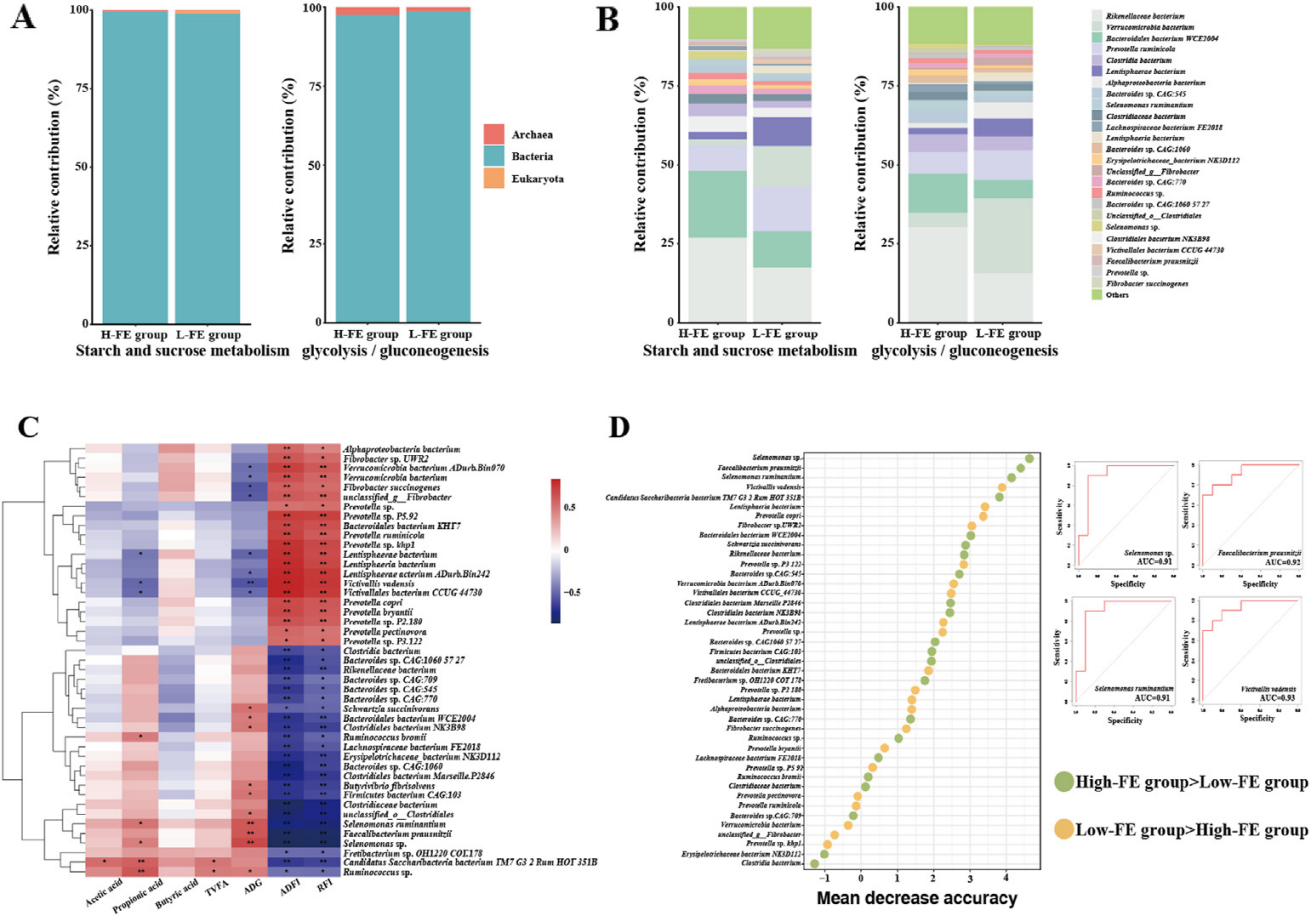
Interactions between different rumen microbiota, functions and phenotypic traits. (A) Contribution analysis for the rumen microbiota in cellulose degradation pathways (domain level). (B) Contribution analysis for significantly different bacteria in cellulose degradation pathways (species level). (C) Spearman's rank correlations between significantly different bacteria and phenotypic traits. (D) Random forest analysis for significantly different bacteria in the rumen between High-FE and Low-FE groups (species level). Low-FE = extreme individual sheep with the highest RFI; High-FE = extreme individual sheep with the lowest RFI; ADG = average daily gain; ADFI = average daily feed intake; TVFA = total volatile fatty acids; RFI = residual feed intake. Significant correlations are shown with ∗ (*P* < 0.05) and ∗∗ (*P* < 0.01).

According to the contribution analysis, the functions of “starch and sucrose metabolism” and “glycolysis/gluconeogenesis” came from the bacteria in the rumen (Fig. 4A). Thus, we further analysed the function of significantly different bacteria by contribution analysis (Fig. 4B). According to the contribution analysis results, the bacteria with the highest contribution to “starch and sucrose metabolism” were *Rikenellaceae* *bacterium*, *Bacteroidales* *bacterium WCE2004* and *Prevotella ruminantium*. However, the highest contributing bacteria in “glycolysis/gluconeogenesis” were different in Low-FE and High-FE groups. The bacterium with the highest contribution in High-FE was *Rikenellaceae* *bacterium,* while the bacterium with the highest contribution in Low-FE was *Verrucomicrobia* *bacterium*.

These bacteria with significantly different abundances in the rumen were used in Spearman correlation analysis with the phenotype to select the key bacteria for the effect of FE in sheep. *Selenomonas* sp. (*r* = −0.83), *Selenomonas ruminantium* (*r* = −0.77), *Faecalibacterium prausnitzii* (*r* = −0.82) and *Victivallis vadensis* (*r* = 0.76) had the highest correlation coefficients with RFI in all bacteria, and these bacteria were considered efficiency-associated bacteria based on Spearman correlation (*P* < 0.01) (Fig. 4C). Moreover, bacteria with significantly different abundances were used to predict FE using a random forest model. Four bacteria with significantly different abundances, including *Selenomonas* sp., *S. ruminantium*, *F. prausnitzii* and *V. vadensis*, were selected by random forest with the highest mean decrease accuracy (MDA) value, with high accuracy (AUC >  0.90), and the resulting random forest model corresponded to the resulting Spearman correlation (Fig. 4D).

### 3.6 Profiling of the rumen proteome

**Fig. 5:**
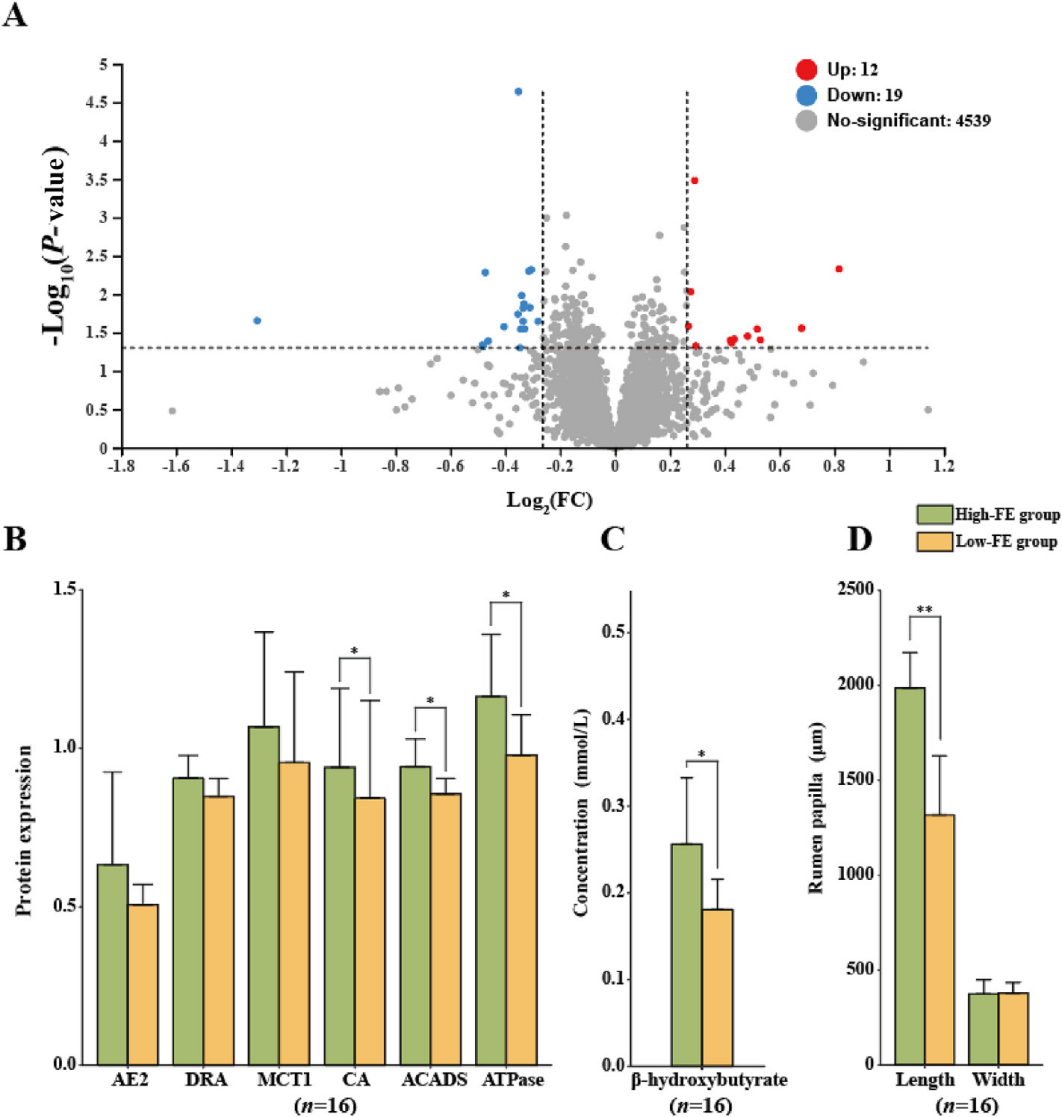
Proteomics data and slices of the rumen epithelium of High-FE and Low-FE groups. (A) Volcano plot of proteins in the rumen epithelium. (B) Protein expression comparison in the rumen epithelium between High-FE and Low-FE groups. (C) The concentration of β-hydroxybutyrate in serum between High-FE and Low-FE groups. (D) The observation results of rumen papillae between High-FE and Low-FE groups. Low-FE = extreme individual sheep with the highest RFI; High-FE = extreme individual sheep with the lowest RFI; RFI = residual feed intake; AE2 = anion exchanger 2; DRA = downregulated in adenoma; MCT1 = monocarboxylate transporter 1; CA = carbonic anhydrase; ACADS = butyryl-CoA dehydrogenase; ATPase = Na^+^/K^+^-ATPase. The statistical models were the *t*-test (normally distributed data) and Wilcoxon test (non-normally distributed data). Significant correlations are showed with ∗ (*P* < 0.05) and ∗∗ (*P* < 0.01).

Proteomics analysis generated a total of 19,536 proteins and identified 4570 proteins by database analysis. There were 31 differentially expressed proteins in the rumen of extreme individual sheep, and 12 and 19 proteins were up- and downregulated in the High-FE group, respectively (Fig. 5A). The transfer protein of VFA did not show significant differences in expression in extreme individual sheep, including anion exchanger 2 (AE2), downregulated in adenoma (DRA), and monocarboxylate transporter 1 (MCT1). However, carbonic anhydrase (CA) was significantly higher in High-FE group than in Low-FE group (*P* = 0.039) (Fig. 5B). We also found that the levels of butyryl-CoA dehydrogenase (ACADS, EC: 1.3.8.1) (|log_2_(FC)| ≥ 0 and *P* = 0.033) and Na^+^/K^+^-ATPase (ATPase) (|log_2_(FC)| ≥ 0 and *P* = 0.044) were significantly higher in High-FE group (Fig. 5B). We further tested the metabolites in serum samples and found that the concentration of β-hydroxybutyrate in serum was significantly higher in the High-FE group than in the Low-FE group (*P* = 0.014) (Fig. 5C). In addition, HE staining of the rumen epithelium showed that the length of the rumen papilla was highly significantly increased in High-FE group (*P* < 0.001). However, the width of the rumen papilla showed no significant difference between groups (*P* = 0.892) (Fig. 5D).

### 3.7 Profiling the liver transcriptome

**Fig. 6:**
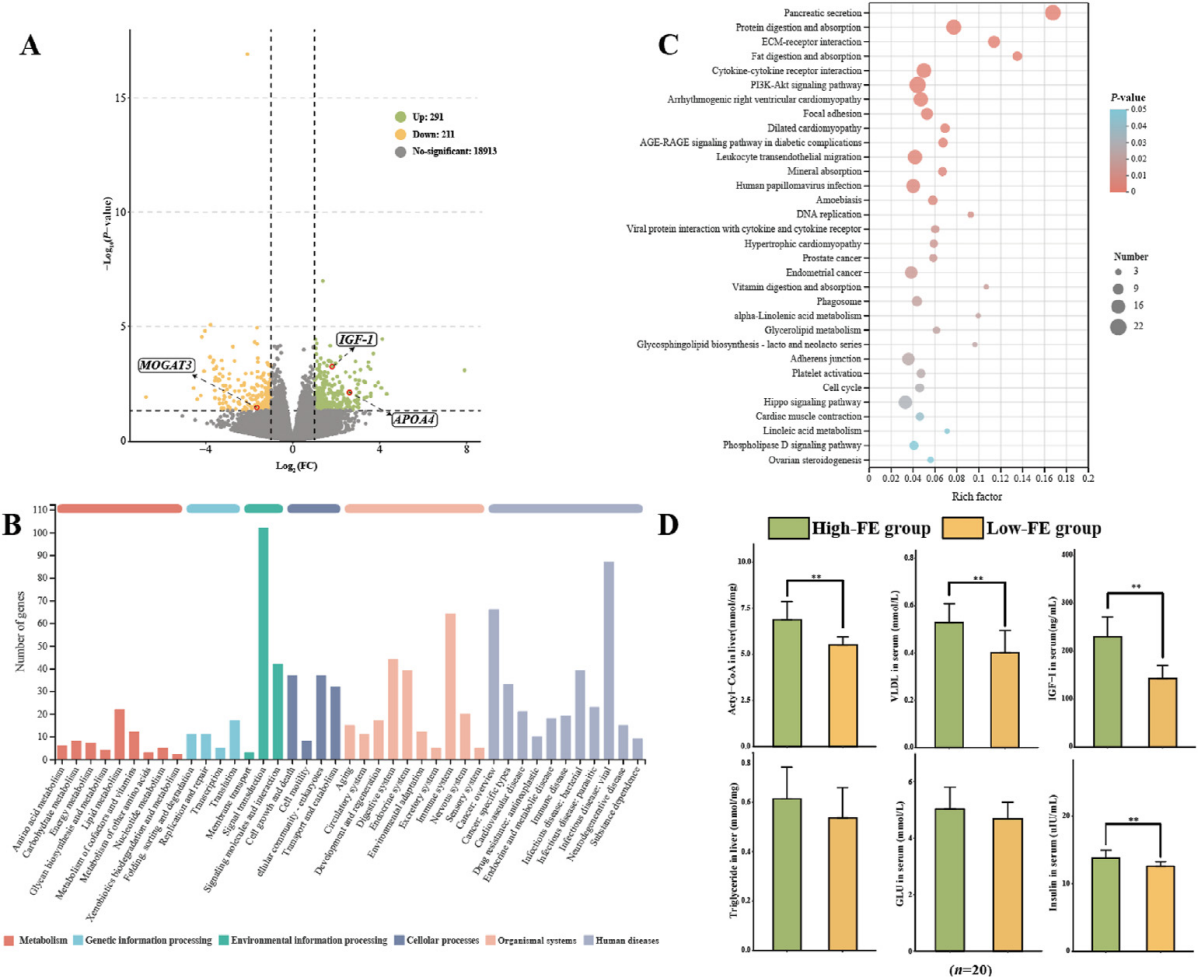
The resulting liver transcriptomics and metabolite data in serum with comparison between High-FE and Low-FE groups. (A) Volcano plot of the liver transcriptome. (B) Functional annotation analysis by KEGG in the liver. (C) The resulting significantly different pathways in the liver compared between High-FE and Low-FE groups. (D) The concentration of metabolites in liver and serum compared between High-FE and Low-FE groups. Low-FE = extreme individual sheep with the highest RFI; High-FE = extreme individual sheep with the lowest RFI; RFI = residual feed intake; IGF-1 = insulin-like growth factor-1; VLDL = very low-density lipoprotein; GLU = glucose; FC = fold change; *APOA4* = apolipoprotein A4; *MOGAT3* = monoacylglycerol O-acyltransferase 3. The statistical models were the *t*-test (normally distributed data) and Wilcoxon test (non-normally distributed data). Significant correlations are shown with ∗ (*P* < 0.05) and ∗∗ (*P* < 0.01).

Transcriptome sequencing generated a total of 127.25 GB of clean reads, and the clean data of each sample reached more than 6 GB. The quality control results of every sample are shown in Table S3. There were 502 DEG in the liver of test group sheep, and 291 and 211 genes were up- and downregulated, respectively, in the High-FE group (Fig. 6A). Most genes were related to lipid metabolism in all metabolic pathways (Fig. 6B). To gain insight into the function of DEG detected in terms of |log_2_ (FC) | ≥ 1 and *P* < 0.05, we carried out KEGG term enrichment analysis for up- and downregulated genes. Thirty-two pathways were significantly enriched, and there were many lipid metabolites in all metabolism pathways (Fig. 6C). We observed downregulation of the monoacylglycerol O-acyltransferase 3 (*MOGAT3*) gene (*P* = 0.037), which is related to triglyceride synthesis, and upregulation of the apolipoprotein A4 (*APOA4*) gene (*P* = 0.005), which is related to VLDL synthesis in the fat digestion and absorption pathway. Moreover, we also noticed *IGF-1* gene (*P* < 0.001) upregulation in the phosphatidylinositide 3-kinases (PI3K-Akt) signalling pathway (Fig. 6A).

We further tested different metabolites and hormones in the serum and liver of all extreme individual sheep (Fig. 6D). The concentrations of acetyl-CoA (*P* = 0.002) in the liver and IGF-1 (*P* = 0.003), insulin (*P* = 0.009) and VLDL (*P* = 0.002) in the serum were highly significantly higher in the High-FE group than in the Low-FE group. However, the concentrations of triglyceride (*P* = 0.241) and GLU (*P* = 0.263) in the serum were not significantly different between the different groups of sheep.

## 4 Discussion

### 4.1 Degradation of cellulose by the rumen microbiota

By integrating microbiota metagenomics, liver transcriptomics and rumen proteomics data, we investigated the contribution of the rumen microbiota and host metabolic mechanisms to the FE of sheep. The rumen is the main organ where digestion of crude fibre occurs, and the digestion process occurs mainly through fermentation by the rumen microbiota. Moreover, Li and Guan (2017) proposed that the RFI of ruminants was related to the fermentation and digestion of the microbiota in the rumen. The degradation of cellulose by rumen microbiota mainly depends on the action of different cellulases encoded by themselves (Gong et al., 2012). From the results, the genes encoding CAZymes were different, which might cause differences in the digestibility of crude fibre in the rumen. The feed enters the reticulum after fermentation by the rumen microbiome; therefore, we further collected digesta in the rumen and reticulum to evaluate the digestibility of crude fibre in different groups of sheep. By comparing NDF contents of digesta in the rumen and reticulum, we found that the rumen microbiota in High-FE group showed a better ability to degrade dietary crude fibre (Fig. S2).

In this study, *Rikenellaceae* *bacterium* mainly contributed to the fermentation process of cellobiose. However, *V.* *bacterium*, rather than *Rikenellaceae* *bacterium,* mainly contributed to the glycolysis/gluconeogenesis pathway in the Low-FE group. Due to changes in substrates in the environment, substitution occurs between microbiomes in the same niche, but their ability to utilize substrates differs (Olson et al., 2017; Seshadri et al., 2018). Thus, the use of *V.* *bacterium* instead of *Rikenellaceae* *bacterium* may lead to differences in the efficiency of pyruvate production, which is the main substrate for VFA, and the different concentrations of TVFA in the rumen also confirmed that there were different utilization efficiencies of cellobioses of the rumen microbiota in different FE sheep. Many previous studies have shown that *Ruminococcus*, *Oscillospira* and *Selenomonas* are the main bacterial genera of the rumen that affect the phenotype of FE (Liu et al., 2022). We also noticed that the species *Selenomonas* sp., *S. ruminantium* of *Selenomonas*, *V. vadensis* of *Victivallis* and *F. prausnitzii* of *Ruminococcus* were the main bacteria affecting the phenotype of RFI.

**Fig. 7:**
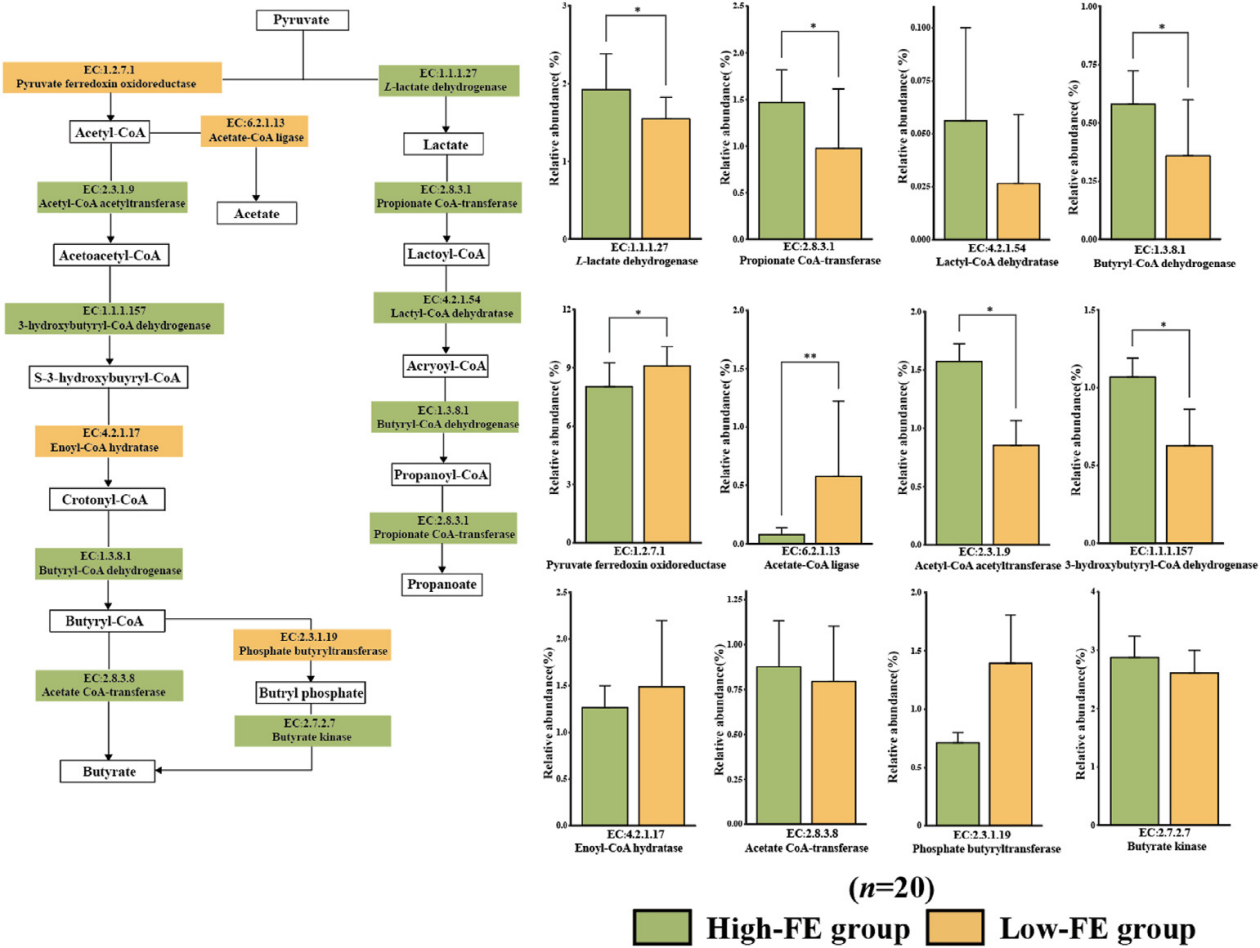
The metabolic pathways of pyruvate fermentation to acetate, propionate and butyrate in rumen between High-FE and Low-FE groups. Low-FE = extreme individual sheep with the highest RFI; High-FE = extreme individual sheep with the lowest RFI; RFI = residual feed intake. The statistical models were the *t*-test (normally distributed data) and Wilcoxon test (non-normally distributed data). Significant correlations are shown with ∗ (*P* < 0.05) and ∗∗ (*P* < 0.01).

Previous studies reported that *S. ruminantium* had the ability to degrade lactic acid in the environment into propionate by D-lactate dehydrogenase and that the cellulose degradation efficiency of cellulose-degrading bacteria in the rumen could be improved by using succinic acid and fibre oligosaccharides (Asanuma and Hino, 2005; Sawanon et al., 2011). *F. prausnitzii* is an important butyrate-producing bacterium whose main fermentation pathway is the butyryl-CoA:acetate-CoA transferase pathway; thus, the concentration of acetate in the rumen is a key driving force for butyrate production (Duncan et al., 2002, 2004; Louis et al., 2010). However, *V. vadensis*, which was enriched in abundance in Low-FE group, could degrade cellobiose to produce acetate and ethanol (Zoetendal et al., 2003). Based on the observation of bacteria in the rumen, we could further conclude the reason for acetate:propionate ratio in the rumen by microbial fermentation in the rumen of different RFI sheep. The rumen microbiota in High-FE group could be more inclined to produce propionate by *S. ruminantium* and *Selenomonas* sp. and consume acetate in the rumen to produce butyrate by *Faecalibacterium prausnitzii*, while the rumen microbiota in Low-FE group tended to produce acetate by *V. vadensis*. The phenotypic indicators of acetate:propionate ratio and the concentration of butyrate in the rumen also proved this result. We further explored the pathways of glycolysis/gluconeogenesis (ko00010), propanoate metabolism (ko00640) and butanoate metabolism (ko00650) by the KEGG database (Fig. 7). The relative abundance of the gene encoding the enzyme producing propionate and acetyl-CoA acetyltransferase (EC: 2.3.1.9) was significantly higher in High-FE group than in Low-FE group. Moreover, the levels of pyruvate ferredoxin oxidoreductase (EC: 1.2.7.1) and acetyl-CoA ligase (EC: 6.2.1.13) were significantly higher in Low-FE group. We also observed that the level of acetate CoA-transferase (EC: 2.8.3.8) was higher in High-FE group than in Low-FE group, proving that rumen bacteria in High-FE group could be more inclined to consume acetate to produce butyrate. The functional prediction of the microbiota in the rumen by KEGG further demonstrated the selection results of key bacteria and further explained the phenotype of VFA.

The types of VFA in the rumen have different contributions to digestion in sheep. Acetate, propionate, and butyrate can generate 62%, 109% and 78% energy (2805 kJ/mol), respectively, compared to the energy produced through the oxidative decomposition of GLU (Ryle and Ørskov, 1990). Therefore, a decrease in acetate:propionate ratio was more favourable for the growth of sheep (Ferreira et al., 2021). By integrating the microbiota metagenome and metabolites, we explored the impact mechanism of FE sheep from the perspective of the rumen microbiota. However, VFA need further uptake and transformation before they can be used by the host. Thus, the absorption and digestion ability of the host was also a key influencing factor for FE.

### 4.2 Nutrient transformation and absorption of the host

The rumen is the main VFA absorption organ of ruminants, and the two types of carrier proteins that transport VFA in the rumen epithelium: VFA^-^/HCO_3_^-^ exchange carrier proteins (DRA, AE2) and VFA^-^/HCO_3_^-^ cotransporters (MCT1) (Aschenbach et al., 2011). In addition, the development of rumen papillae is also an important influencing factor, providing a larger contact area for VFA^-^. The results indicate that the efficiency of VFA^-^ absorption by the rumen epithelium in High-FE group is predominantly influenced by rumen papillae. In addition, membrane proteins (Na^+^/K^+^-ATPase) establishing concentration gradients also affect substrate transporter activity, and CA plays a role in maintaining intracellular pH and the diffusion gradient across the plasma membrane for VFA uptake (Bond et al., 2019). The concentration of HCO_3_^-^ in rumen epithelial cells drives carrier protein exchange of VFA^-^ and HCO_3_^-^. CA can increase the exchangers VFA^-^ and HCO_3_^-^ in rumen epithelial cells by enhancing the synthesis efficiency of HCO_3_^-^ (Aschenbach et al., 2009), and could affect the pH of the rumen.

Giesecke et al. (1979) reported that β-hydroxybutyrate is the main source of energy for rumen epithelial cells, and 80% of butyrate is converted to β-hydroxybutyrate in the rumen epithelium, motivating us to explore the pathway of synthesizing butyrate from β-hydroxybutyrate in the ruminal epithelium by KEGG (ko00650). We found different levels of butyryl-CoA dehydrogenase in sheep with different RFI. Butyryl-CoA dehydrogenase catalyses butyrate to crotonyl CoA, which is the first step in the pathway of butyrate from β-hydroxybutyrate in the rumen epithelium. In addition, the concentration of β-hydroxybutyrate in serum also showed that the rumen epithelium can more efficiently utilize butyrate to form β-hydroxybutyrate in High-FE group.

The liver is a key organ for the entire metabolic system for ruminants; it further converts VFA and ketones coming from the rumen into metabolites that are suitable for absorption (Armentano, 1992). The propionate that is absorbed into the blood is metabolized in the liver for gluconeogenesis (Aschenbach et al., 2010). The liver is the main organ that converts volatile acids into absorbable substances in the sheep body, especially the gluconeogenesis pathway. However, there were no DEG in the gluconeogenesis pathway in the current study. From the results of insulin and GLU levels, we speculated that the liver of High-FE group could be affected by insulin to avoid the excess production of glucose by gluconeogenesis in response to the increased propionate provided from the rumen.

Most microbiome-derived acetate is absorbed directly into the blood, and some is converted to acetyl-CoA in the liver (Zhang et al., 2021). In the current study, more acetate induced concentration increases of acetyl-CoA in the liver of High-FE group. The difference in acetyl-CoA levels in the liver could be the reason why many lipid metabolism pathways were enriched in the liver transcriptome. Liver transcriptomics analysis revealed low expression of *MOGAT3* in High-FE group, members of the acylglycerol O-acyltransferase (*DGAT2*/*MOGAT*) gene family that are involved in the synthesis of diacylglyceride (DAG) and triacylglyceride from monoacylglyceride (Jiang et al., 2014). In addition, triglycerides are converted into lipoproteins by apolipoprotein A and enter the blood. *APOA4* regulates the process of VLDL production and entry into the blood in the liver. Upregulation of *APOA4* in the liver of High-FE group increases the ability of fat to enter the blood from the liver. Fujimoto et al. (1992) reported that the feeding intake of test mice was decreased by injecting chylous solution into the vein, and this result vanished by removing the APOA4 protein, proving that APOA4 protein in VLDL could decrease feeding intake. Glatazle et al. (2004) found that APOA4 protein binding cholecystokinin-1 receptor (CCK-1R) can inhibit gastric activity through the vagus nerve. Therefore, the resulting upregulation of *APOA4* in the liver could indirectly influence RFI by feeding intake. Further measurement of metabolites in the liver and serum proved that the high-FE liver reduced the storage of lipids by downregulating triglyceride synthesis and upregulating VLDL synthesis and secretion. Most IGF-1 is secreted by the liver and stimulates the growth of bone and muscle (Laron, 2001). Previous studies revealed that low-RFI lambs had higher circulating IGF-1 (Moore et al., 2005; Montelli et al., 2021). In combination with changes in feed intake, different FE traits in sheep can be determined. The liver transcriptome and metabolites in serum showed that the liver is an important organ that influences FE by regulating triglyceride synthesis and IGF-1 secretion.

**Fig. 8:**
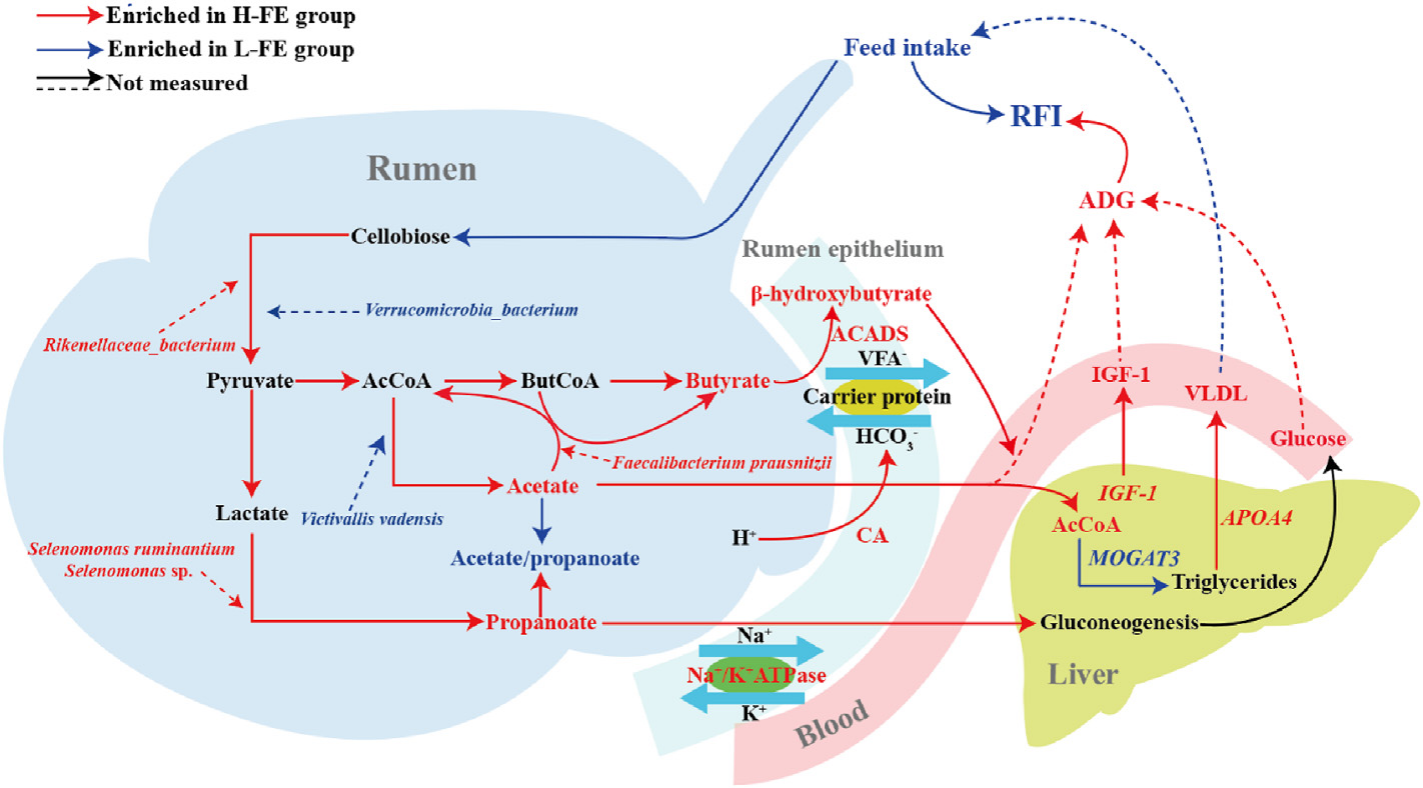
The influence mechanism by which the combined action of the host and rumen microbiota impact FE in sheep. Low-FE = extreme individual sheep with the highest RFI; High-FE = extreme individual sheep with the lowest RFI; RFI = residual feed intake; ADG = average daily gain; CA = carbonic anhydrase; ACADS = butyryl-CoA dehydrogenase; IGF-1 = insulin-like growth factor-1; VLDL = very low-density lipoprotein; *MOGAT3* = monoacylglycerol O-acyltransferase 3; *APOA4* = apolipoprotein A4; AcCoA = acetyl-CoA; ButCoA = butyryl-CoA; VFA = volatile fatty acids.

In summary, we have elucidated the collaborative mechanisms between the host and its rumen microbiota and how they influence feed efficiency in sheep (Fig. 8). As depicted, the rumen microbiota in High-FE group more effectively utilizes cellulose, converting it into propionate and metabolizing acetate into butyrate, which provides the rumen epithelium with more energy substances. Simultaneously, the host indirectly enhances the transport efficiency of VFA transport proteins by regulating HCO_3_^-^. After VFA enter the liver, the metabolites produced by regulating hepatic lipid metabolism, along with IGF-1, ultimately influence the host's RFI phenotype. These findings indicate that the varied feed efficiency in sheep is determined by the combined action of the host and rumen microbiota.

## 5 Conclusion

The findings of this study provide an understanding of the mechanism by which the combined action of the host and rumen microbiota impacts FE in sheep. *F. prausnitzii, S. ruminantium, Selenomonas* sp., and *Rikenellaceae* *bacterium*, improved the utilization of crude fibre and VFA for host animals. Higher VFA uptake via the absorption capacity of the rumen epithelium and rumen microbiota increases the accumulation of acetyl-CoA in the liver which affects triglyceride synthesis and VLDL secretion and subsequently decreases ADFI in the host. In addition, higher nutrient transformation and regulation of IGF-1 increased ADG in the host, combined with ADFI, finally determining the phenotype of FE.

## Author contributions

**Guangchen Zhou:** Conceptualization, Methodology, Formal analysis, Writing- Original draft preparation. **Juda Li:** Investigation, Data curation. **Xuhui Liang:** Investigation, Visualization. **Bohua Yang:** Methodology. **Ximeng He:** Investigation, Data curation. **Hongyu Tang:** Methodology. **Hongran Guo:** Methodology. **Gongwei Liu:** Software. **Wenyuan Cui:** Supervision. **Yulin Chen:** Conceptualization, Funding acquisition. **Yuxin Yang:** Conceptualization, Methodology, Funding acquisition, Writing- Reviewing and Editing.

## Declaration of competing interest

We declare that we have no financial or personal relationships with other people or organizations that can inappropriately influence our work, and there is no professional or other personal interest of any nature or kind in any product, service and/or company that could be construed as influencing the content of this paper.
