# Supplementary material for: Multi-omics revealed the mechanism of feed efficiency in sheep by the combined action of the host and rumen microbiota
Source: Anim Nutr. 2024 Apr 27;18:367–79. doi: 10.1016/j.aninu.2024.04.009 (PMC11406083; doi:10.1016/j.aninu.2024.04.009)
Supplement: Multimedia component 1 [file mmc1.docx]

**Availability of data and materials**

The rumen metagenome sequences were deposited into the NCBI Sequence Read Archive (SRA) under the accession number SUB12140508. The RNA-Seq data were deposited into NCBI SRA under the accession number SUB12160861. The proteomics sequences were deposited into iProX under the accession number IPX0005475000.

**Table S1** The quality of metagenome

| Sample | Clean reads | Optimized reads | Contigs | N50, bp | N90, bp | ORF | Total length, bp | Average length, bp |
| --- | --- | --- | --- | --- | --- | --- | --- | --- |
| H1 | 71,491,074 | 54,424,968 | 822,683 | 642 | 349 | 1,043,101 | 476,975,052 | 457.27 |
| H2 | 59,280,960 | 38,683,096 | 665,168 | 519 | 334 | 838,368 | 316,278,927 | 377.26 |
| H3 | 71,595,362 | 51,997,210 | 781,790 | 684 | 353 | 1,019,102 | 474,392,265 | 465.50 |
| H4 | 64,339,720 | 44,197,696 | 824,386 | 557 | 340 | 1,043,559 | 414,640,929 | 397.33 |
| H5 | 65,990,354 | 47,074,018 | 699,480 | 743 | 358 | 942,411 | 439,591,782 | 466.45 |
| H6 | 74,752,488 | 50,541,414 | 904,624 | 602 | 346 | 1,142,403 | 500,940,828 | 438.50 |
| H7 | 74,301,174 | 55,861,600 | 889,189 | 683 | 353 | 1,148,988 | 538,219,020 | 468.43 |
| H8 | 66,318,714 | 50,433,730 | 646,272 | 729 | 359 | 848,186 | 412,047,138 | 485.80 |
| H9 | 76,308,662 | 56,929,492 | 824,707 | 659 | 350 | 1,059,827 | 490,417,881 | 462.73 |
| H10 | 65,524,512 | 50,084,068 | 732,489 | 661 | 351 | 935,957 | 436,907,349 | 466.80 |
| L1 | 56,358,796 | 39,722,296 | 658,658 | 754 | 369 | 838,465 | 428,211,099 | 510.71 |
| L2 | 53,697,722 | 39,324,158 | 629,589 | 739 | 363 | 807,552 | 398,554,305 | 493.53 |
| L3 | 56,785,388 | 43,475,924 | 681,305 | 653 | 353 | 844,198 | 396,434,562 | 469.60 |
| L4 | 54,637,400 | 39,441,964 | 619,298 | 674 | 360 | 761,336 | 368,418,144 | 483.91 |
| L5 | 68,039,544 | 51,641,976 | 789,841 | 675 | 352 | 1,011,171 | 474,415,380 | 469.17 |
| L6 | 62,825,494 | 48,569,596 | 777,383 | 730 | 365 | 992,565 | 488,262,252 | 491.92 |
| L7 | 56,864,866 | 44,011,734 | 768,685 | 627 | 352 | 959,465 | 437,687,637 | 456.18 |
| L8 | 63,977,656 | 50,151,462 | 786,487 | 739 | 361 | 1,047,006 | 505,426,194 | 482.73 |
| L9 | 65,492,992 | 48,339,144 | 732,443 | 666 | 350 | 943,796 | 438,029,532 | 464.11 |
| L10 | 82,432,538 | 64,076,954 | 869,189 | 725 | 357 | 1,145,036 | 549,186,657 | 479.62 |

ORF=open reading frame.

**Table S2** The relative abundance of glycoside hydrolase in rumen (%)

| Item | GHs | High-FE group | Low-FE group | *P*-value |
| --- | --- | --- | --- | --- |
| β-glucosidase | GH1 | 0.28±0.083 | 0.14±0.099 | 0.003 |
|  | GH3 | 2.64±0.319 | 2.01±0.575 | 0.009 |
|  | GH5 | 0.19±0.050 | 0.35±0.099 | 0.001 |
|  |  |  |  |  |
| Exoglucanase | GH5 | 0.19±0.050 | 0.35±0.099 | 0.001 |
|  |  |  |  |  |
| Endoglucanase | GH5 | 0.19±0.050 | 0.35±0.099 | 0.001 |
|  | GH74 | 0.09±0.021 | 0.13±0.028 | 0.003 |
|  |  |  |  |  |
| Xylanase | GH141 | 0.20±0.036 | 0.34±0.110 | 0.002 |
|  | GH10 | 0.72±0.128 | 0.91±0.172 | 0.011 |
|  | GH3 | 2.64±0.319 | 2.01±0.575 | 0.009 |
|  | GH1 | 0.28±0.083 | 0.14±0.099 | 0.003 |

Low-FE=extreme individual sheep with the highest RFI; High-FE=extreme individual sheep with the lowest RFI; GH= glycoside hydrolases.

**Table S3** The quality of RNA-Seq

| Group | Sample | Raw reads | Clean reads | Error rate, % | Q20, % | Q30, % | GC content, % |  |
| --- | --- | --- | --- | --- | --- | --- | --- | --- |
|  |  |  |  |  |  |  |  |  |
| Low-FE | liverL10 | 44,440,242 | 43,980,116 | 0.0251 | 98.04 | 94.04 | 42.08 |  |
|  | liverL9 | 42,973,870 | 42,537,012 | 0.0249 | 98.08 | 94.17 | 41.79 |  |
|  | liverL8 | 43,818,076 | 43,310,758 | 0.0256 | 97.78 | 93.51 | 42.04 |  |
|  | liverL7 | 44,918,936 | 44,498,588 | 0.0249 | 98.05 | 94.32 | 48.50 |  |
|  | liverL6 | 43,645,162 | 43,163,744 | 0.0249 | 98.07 | 94.29 | 46.06 |  |
|  | liverL5 | 42,188,882 | 41,689,512 | 0.0249 | 98.09 | 94.27 | 44.37 |  |
|  | liverL4 | 43,141,402 | 42,639,914 | 0.0251 | 97.98 | 94.10 | 45.12 |  |
|  | liverL3 | 44,202,022 | 43,655,716 | 0.0252 | 97.96 | 93.98 | 44.04 |  |
|  | liverL2 | 44,220,022 | 43,678,134 | 0.0249 | 98.05 | 94.23 | 45.16 |  |
|  | liverL1 | 41,149,088 | 40,640,858 | 0.0253 | 97.9 | 93.84 | 44.31 |  |
|  |  |  |  |  |  |  |  |  |
| High-FE | liverH10 | 45,473,730 | 44,758,238 | 0.0251 | 98.00 | 94.09 | 42.73 |  |
|  | liverH9 | 45,628,418 | 44,912,858 | 0.0244 | 98.20 | 94.75 | 49.77 |  |
|  | liverH8 | 46,250,984 | 45,314,182 | 0.0250 | 97.99 | 94.18 | 44.69 |  |
|  | liverH7 | 46,204,508 | 45,301,618 | 0.0247 | 98.10 | 94.50 | 47.82 |  |
|  | liverH6 | 44,881,036 | 44,401,166 | 0.0247 | 98.10 | 94.43 | 47.71 |  |
|  | liverH5 | 46,628,638 | 45,913,550 | 0.0246 | 98.17 | 94.60 | 46.21 |  |
|  | liverH4 | 44,862,520 | 44,217,496 | 0.0247 | 98.12 | 94.45 | 45.54 |  |
|  | liverH3 | 46,302,412 | 45,602,234 | 0.0249 | 98.03 | 94.28 | 45.82 |  |
|  | liverH2 | 45,970,608 | 45,268,558 | 0.0250 | 98.04 | 94.17 | 42.56 |  |
|  | liverH1 | 46,396,722 | 45,777,156 | 0.0248 | 98.03 | 94.39 | 50.28 |  |

Low-FE=extreme individual sheep with the highest RFI; High-FE=extreme individual sheep with the lowest RFI;


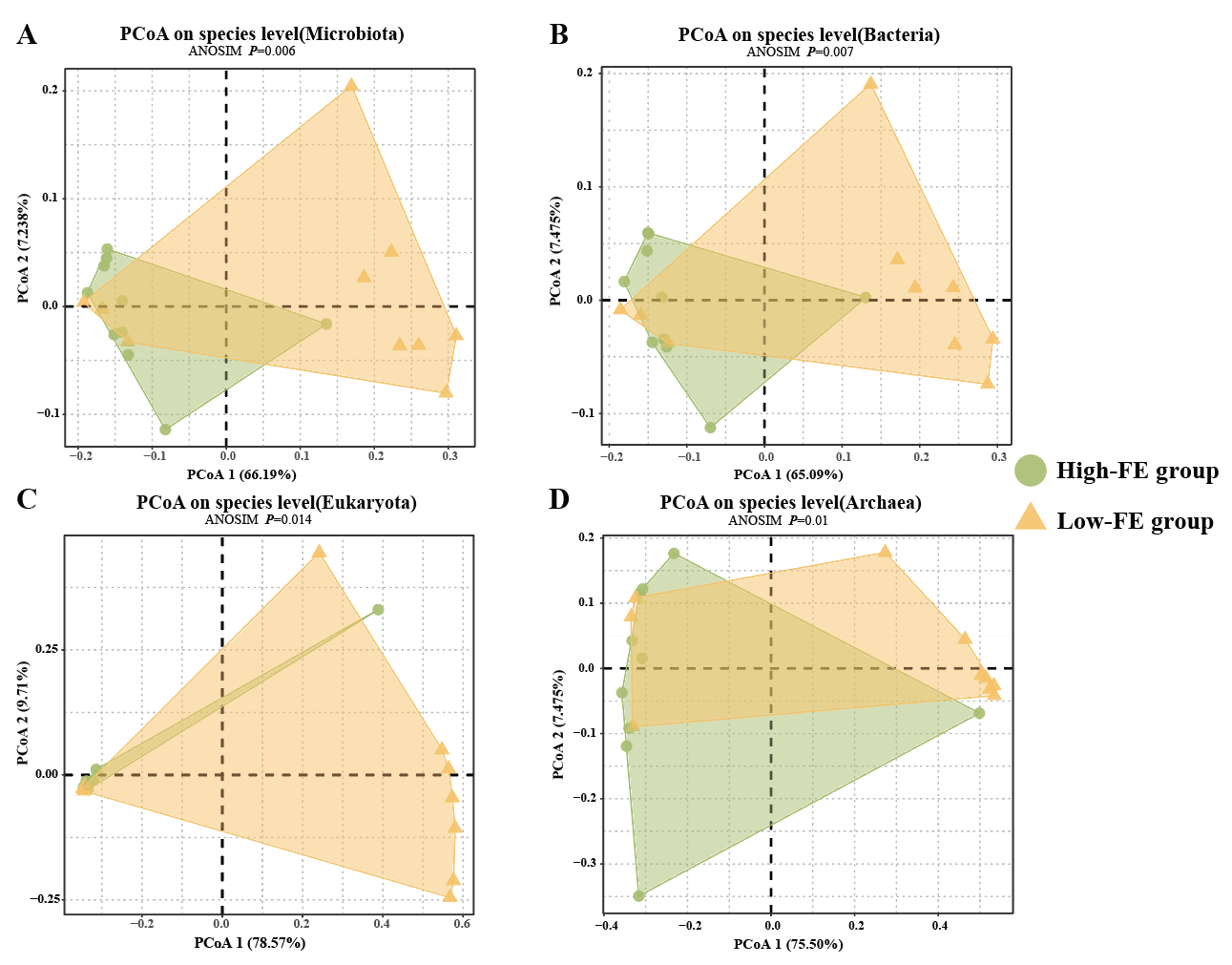


**Fig. S1** Principal coordinates analysis (PCoA) profile of microbial diversity based on Bray‒Curtis distance matrixes. (A) Microbiota; (B) Bacteria; (C) Eukaryotes; (D) Archaea. Low-FE=extreme individual sheep with the highest RFI; High-FE=extreme individual sheep with the lowest RFI.


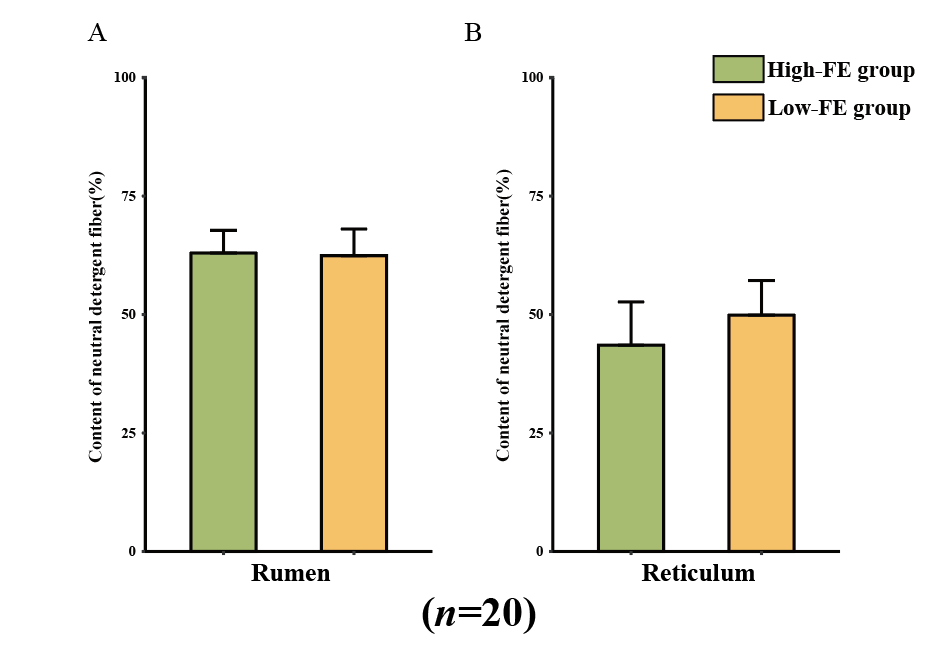


**Fig.S2** The content of neutral detergent fibre (NDF) in chyme between High-FE and Low-FE groups. (A) Rumen; (B): Reticulum. Low-FE=extreme individual sheep with the highest RFI; High-FE=extreme individual sheep with the lowest RFI.
